# International growth of individual placement and support

**DOI:** 10.1017/S2045796020000955

**Published:** 2020-11-13

**Authors:** G. R. Bond, H. Lockett, J. van Weeghel

**Affiliations:** 1 Westat, Lebanon, NH, USA; 2 The University of Auckland, Auckland, New Zealand; 3 Wise Group, Hamilton, New Zealand; 4 Phrenos Center of Expertise, Utrecht, the Netherlands; 5 Tilburg University, Tilburg, the Netherlands

## Introduction

Why are some evidence-based practices (EBPs) more widely disseminated worldwide than others? It typically takes nearly two decades for an intervention to be adopted in routine practice after research demonstrates its effectiveness, even *within* the country where it originates (Green *et al*., 2009). Transporting EBPs to other countries adds further difficulties and often is unsuccessful (Burns, 2000). One widely implemented EBP is individual placement and support (IPS), an employment programme for adults with serious mental illness developed in the USA that has spread to four continents over the past two decades (Drake *et al*., 2020). This essay discusses factors affecting expansion of IPS around the globe.

In the 1990s, Deborah Becker and Robert Drake developed IPS and demonstrated its effectiveness in a rural community mental health centre in the USA. IPS is based on eight principles: focus on open employment, zero exclusion and eligibility based on service user choice, attention to service user preferences, rapid job search, integration of employment services and mental health treatment, personalised benefits counselling, targeted job development and individualised, long-term support. A practitioner manual (Swanson and Becker, 2018), online courses (ipsworks.org/index.php/training-courses/), a certification process for IPS specialists, and a well-validated fidelity scale (Lockett *et al*., 2016) promote adherence to model standards wherever it is implemented. Since its inception, researchers have continuously conducted rigorous research on IPS. Among 28 randomised controlled trials (RCTs) comparing IPS to traditional vocational services (16 conducted outside the USA), all but one have shown superior employment outcomes for IPS (Bond *et al*., 2020).

IPS has grown rapidly within the USA since the establishment in 2002 of the IPS Learning Community, guided by the IPS Employment Center, which coordinates education, training, technical assistance, fidelity and outcome monitoring and regular communications through newsletters, bimonthly calls and annual meetings. By 2020, the learning community membership had grown to 366 IPS teams in 24 states in the USA and over 100 teams in six other countries (Drake *et al*., 2020).

## International growth

IPS programmes are currently found in 20 countries – 7 in the learning community (USA, Netherlands, Italy, Spain, UK, Canada and New Zealand) and 13 others (Australia, Belgium, China, Czech Republic, Denmark, France, Germany, Iceland, Ireland, Japan, Norway, Sweden and Switzerland). Some countries have dozens of IPS programmes (UK and Netherlands), and others have very few (Iceland and Belgium). The count of IPS programmes worldwide is unknown.

In each country, the organisation of governmental services, funding of mental health and vocational services, labour laws, disability policies, history and culture have all shaped the evolution of IPS services. Given this diversity, each country has followed a unique pathway to IPS development (IIMHL, 2019; Drake, 2020). Yet, IPS has spread worldwide in a relatively brief span of time, in most places maintaining adherence to IPS fidelity as the guide to implementation. We hypothesise that several factors contribute to this growth.

## Enablers of international expansion

### Unique features of IPS

Several features differentiate IPS from most other mental health EBPs (Bond *et al*., 2010). The goal of IPS – open employment – explicitly addresses both individual recovery goals and societal priorities. IPS is a pragmatic, easily understood approach. It is flexible, has shown effectiveness in virtually all service user subgroups in which it has been studied, and is well-suited to diverse settings including both rural and urban communities. Because employment outcomes are face valid and easily measured, all stakeholders can understand IPS findings. These and other characteristics make IPS appealing to mental health leaders, government officials and the public at large.

### Local champions

Committed and persistent leaders have often spearheaded spread of IPS in their countries. Widespread acceptance of an innovative programme typically takes years, often with a few determined proponents advocating for it long before national policy has moved towards adoption (Drake, 2020).

### Replications of IPS research outside the USA

In deciding to adopt a new programme model, key decision-makers (e.g. mental health and rehabilitation leaders and government officials) are most persuaded by evaluations conducted locally. For example, the EQOLISE study, an RCT of IPS in six European countries (Burns *et al*., 2007), was influential in counteracting resistance to adopting an intervention developed in the USA, even though US studies showed it was effective. The EQOLISE findings for the Rimini and London sites spurred the expansion of IPS across Northern Italy and facilitated the formation of the UK's IPS Centre of Excellence. In the Netherlands, IPS gained official recognition in mental health guidelines after a successful Dutch multisite RCT (IIMHL, 2019; Drake, 2020).

### Influence of IPS Learning Community

The success of the IPS Learning Community has been an inspiration to IPS advocates outside the USA, modelling effective IPS implementation strategies and the power of peer learning. Furthermore, IPS Employment Center colleagues have fostered international growth by providing encouragement, mentoring and consultation (van Weeghel *et al*., 2020).

### Technical assistance centres

Recognising IPS as a core component of the mental health service system, a half dozen countries have established technical assistance centres to provide training, fidelity reviews and national coordination of IPS programmes. These centres have been crucial for establishing new IPS programmes and maintaining existing ones (Becker and Bond, 2020).

### National initiatives and leadership

Several countries have moved beyond the early adoption phase to formulate ambitious national plans. In England, the National Health Service committed to ‘doubling access to IPS services by 2021’ (Melleney and Kendall, 2020). In the Netherlands, a broad group of mental health providers, vocational rehabilitation and welfare stakeholders, along with service users and their families, adopted a national action plan for the recovery of people with serious mental illness, emphasising that IPS should be accessible to the entire target population (van Weeghel *et al*., 2020).

## Barriers to expansion

Nonetheless, many challenges remain for countries at all stages of IPS adoption – countries where leaders contemplate introducing IPS, countries with early adopters in specific regions and countries aspiring to national expansion. Barriers include those at both service delivery and systems levels.

At the service delivery level, mental health professionals often assume that people on their caseloads are incapable of working in open employment (Bonfils, 2020) and resist integrating employment services within clinical teams (Priest and Lockett, 2020). System barriers include those created by economic and social legislation (Latimer *et al*., 2020). Vocational systems worldwide remain invested in non-EBPs and lack coordination between governmental agencies responsible for health, welfare and employment (Lockett *et al*., 2018).

## Conclusions

We have witnessed wide expansion of IPS in the quarter-century since the publication of the first RCT showing the effectiveness of IPS. To put this in perspective, diffusion of this employment innovation has been more rapid than many noteworthy medical breakthroughs (Drake *et al*., 2008). However, questions remain.

First, what can we expect in this COVID-19 era? We do not know how long this pandemic will continue to affect traditional behaviours, including delivery of mental health services that historically depended on face-to-face contact. Nor do we know the implications for the recovery of national and local economies. On the one hand, in the face of huge governmental deficits and devastated budgets for health care, future prospects for funding IPS services are at risk. On the other hand, with rising mental health issues and unemployment, the need for integrated mental health and employment support through IPS is more urgent than ever.

Second, how do we ensure increased access to IPS without compromising fidelity? Scale-up nearly always requires utilising existing service structures, and for many developed countries this means developing an integration strategy from the available network of government-contracted, free-standing employment agencies. This approach to expansion, which has been applied in the UK and Australia, requires buy-in from both mental health and employment agencies to co-locate and provide on-site technical assistance for high-fidelity implementation (Waghorn *et al*., 2020).

A third unresolved question is, how we bring IPS into routine use? Traditionally, mental healthcare organisations have been the main actors adopting IPS practices. However, because most people with serious mental illness do not have access to mental health treatment (Drake and Essock, 2009), mental health organisations are unlikely to reach the whole target group who could benefit from IPS, nor do they have adequate resources to expand IPS on their own. To attain optimal adoption and sustainability, systems need broad coalitions and shared ownership of IPS. These coalitions need to include, at both local and national levels, mental healthcare providers, generic social and employment services, the vocational rehabilitation system, service user and family organisations and (in some countries) health insurance companies.

In conclusion, the widespread expansion of IPS has not occurred by chance. IPS is a well-defined, easily understood innovation, which responds to the employment aspirations of service users and families. Three decades of rigorous research, national and local leadership and persistence, and the generous sharing of expertise and implementation knowledge globally have facilitated its spread.
